# Germline variants of *ATG7* in familial cholangiocarcinoma alter autophagy and p62

**DOI:** 10.1038/s41598-022-13569-4

**Published:** 2022-06-20

**Authors:** Stephanie U. Greer, Jiamin Chen, Margret H. Ogmundsdottir, Carlos Ayala, Billy T. Lau, Richard Glenn C. Delacruz, Imelda T. Sandoval, Sigrun Kristjansdottir, David A. Jones, Derrick S. Haslem, Robin Romero, Gail Fulde, John M. Bell, Jon G. Jonasson, Eirikur Steingrimsson, Hanlee P. Ji, Lincoln D. Nadauld

**Affiliations:** 1 Division of Oncology, Department of Medicine, Stanford University School of Medicine, Stanford, CA 94305 USA; 2 Department of Anatomy, Faculty of Medicine, BioMedical Center, University of Iceland, Sturlugata 8, 101 Reykjavik, Iceland; 3 Division of General Surgery, Department of Surgery, Stanford University School of Medicine, Stanford, CA 94305 USA; 4 Intermountain Precision Genomics Program, Intermountain Healthcare, Saint George, UT 84790 USA; 5 Oklahoma Medical Research Foundation, Oklahoma University, Oklahoma City, OK 73104 USA; 6 Department of Pathology, Landspítali-University Hospital, 101 Reykjavik, Iceland; 7 Stanford Genome Technology Center, Stanford University, Palo Alto, CA 94304 USA; 8 Faculty of Medicine, University of Iceland, Sturlugata 8, 101 Reykjavik, Iceland; 9 Department of Biochemistry and Molecular Biology, Faculty of Medicine, BioMedical Center, University of Iceland, Sturlugata 8, 101 Reykjavik, Iceland

**Keywords:** Cancer, Genetics

## Abstract

Autophagy is a housekeeping mechanism tasked with eliminating misfolded proteins and damaged organelles to maintain cellular homeostasis. Autophagy deficiency results in increased oxidative stress, DNA damage and chronic cellular injury. Among the core genes in the autophagy machinery, *ATG7* is required for autophagy initiation and autophagosome formation. Based on the analysis of an extended pedigree of familial cholangiocarcinoma, we determined that all affected family members had a novel germline mutation (c.2000C>T p.Arg659* (p.R659*)) in *ATG7*. Somatic deletions of *ATG7* were identified in the tumors of affected individuals. We applied linked-read sequencing to one tumor sample and demonstrated that the *ATG7* somatic deletion and germline mutation were located on distinct alleles, resulting in two hits to *ATG7*. From a parallel population genetic study, we identified a germline polymorphism of *ATG7* (c.1591C>G p.Asp522Glu (p.D522E)) associated with increased risk of cholangiocarcinoma. To characterize the impact of these germline *ATG7* variants on autophagy activity, we developed an *ATG7*-null cell line derived from the human bile duct. The mutant p.R659* ATG7 protein lacked the ability to lipidate its LC3 substrate, leading to complete loss of autophagy and increased p62 levels. Our findings indicate that germline *ATG7* variants have the potential to impact autophagy function with implications for cholangiocarcinoma development.

## Introduction

Autophagy is a conserved cellular pathway tasked with the recycling of misfolded proteins and damaged organelles. Playing a fundamental role in cellular homeostasis, autophagy regulates many different biological functions including the initiation of senescence and maintenance of genome stability. Reflecting its important intracellular function across many organisms, the core set of autophagy related genes (ATG) are highly conserved across mammals. Given their essential role in mammalian cell maintenance, autophagy genes are increasingly being implicated in the pathogenesis of different diseases that include neurodegenerative disorders, autoimmune diseases and cancer^1^.

Cellular stressors can initiate autophagy through various signaling pathways. The core ATG proteins mediate the induction and formation of autophagosomes allowing subsequent fusion with lysosomes and the degradation of autophagosomal cargo^2^. One of the critical steps following autophagy induction is the formation of a double membrane structure, known as the phagophore. Subsequently, lipidated LC3/GABARAPs are added to the phagophore and growing autophagosome where they recruit interacting partners. An example is the autophagy receptor protein p62/SQSTM1, also known as Sequestosome-1. This protein is recruited via a physical interaction to the autophagosome facilitating the degradation of ubiquitinated cargo during autophagy. Importantly, genetic variants in ATG genes have functional implications in autophagy and lead to specific diseases^1^. For example, germline mutations among several autophagy genes, including *ATG5* and *ATG16L1*, have been identified among hereditary disorders involving neurodegeneration and autoimmunity. Although the role of autophagy in cancer remains unclear, SNPs in ATG5 and ATG7 have been associated with clear cell renal cell carcinoma^3^. Presumably, these mutations affect autophagy function.

Cholangiocarcinoma (CCA; MIM: 615619) is an aggressive, difficult-to-treat epithelial carcinoma arising from the bile duct. Once diagnosed, this biliary duct cancer has a dismal five-year survival rate of less than 10%^4,5^. CCAs are categorized into intrahepatic, perihilar and distal subtypes based on their anatomical location. Perihilar CCA, or Klatskin tumor, is anatomically located at the confluence of the right and left hepatic bile ducts, and accounts for approximately 50% of all cases^6^. Genomic studies of CCA have implicated a variety of known cancer drivers such as *TP53*, *KRAS, ERBB2* and others that play a role in oncogenic processes^7–10^.

In this study, we identified a novel germline mutation in *ATG7* (NM_001349232.2: c.2000C>T p.Arg659* (p.R659*)) from a pedigree with familial CCA by comprehensive whole genome and exome sequencing analysis. This germline mutation segregated with affected family members all of whom had perihilar cholangiocarcinoma. Moreover, we detected somatic deletion of *ATG7* and loss of heterozygosity among the cancers in this family. Together, these mutations point to a complete loss of the wildtype alleles of *ATG7*. In addition, we conducted a population study of *ATG7* polymorphisms associated with CCA in the Icelandic population and identified a polymorphic variant of *ATG7* (NM_001349232.2: c.1591C>G p.Asp522Glu (p.D522E)) that was associated with elevated risk of developing this cancer. For this study, we sought to determine the consequences of this mutation and polymorphism.

During autophagy, cytoplasmic materials targeted for degradation are encapsulated in a transient structure called an autophagosome, which subsequently fuses with a lysosome containing digestive enzymes^11^. Lipidated LC3 (LC3-II) is essential for autophagosome maturation and also for the recognition of targeted cargo in selective autophagy^12,13^. The ATG7 protein plays an essential role during autophagosome formation because it is required for two critical conjugation reactions that are part of the multistep process to lipidate LC3. With its E1-like enzymatic activity, ATG7 adenylates LC3-I so that it can be lipidated to generate LC3-II, and also ATG7 adenylates ATG12 as part of the process that generates ATG5-ATG12 conjugates^14,15^. Genetically engineered mouse models initially demonstrated that hepatocyte-specific knockout of *ATG7* resulted in the formation of liver adenomas that did not progress to cancerous tumors^16^. However, a later study found that hepatocyte-specific knockout of *ATG7* resulted in hepatomegaly and hepatocellular carcinoma, suggesting autophagy deficiency might promote tumorigenesis in hepatobiliary tissues^17^.

To study the functional impact of the mutation p.R659* and p.D522E polymorphism on autophagy function we conducted experiments using a human bile duct cell line. Based on the context, we hypothesized that the germline mutation would have greater functional consequences than the polymorphism. Our functional studies demonstrated that the *ATG7* germline mutation (p.R659*) led to a loss of function in autophagy and increased p62 levels in bile duct epithelial cells. The *ATG7* polymorphism (p.D522E) had a less dramatic impact on autophagy function. Overall, these results suggest a potential role of autophagy in CCA development.

## Results

### Identifying a germline *ATG7* mutation associated with hereditary CCA

**Figure 1:**
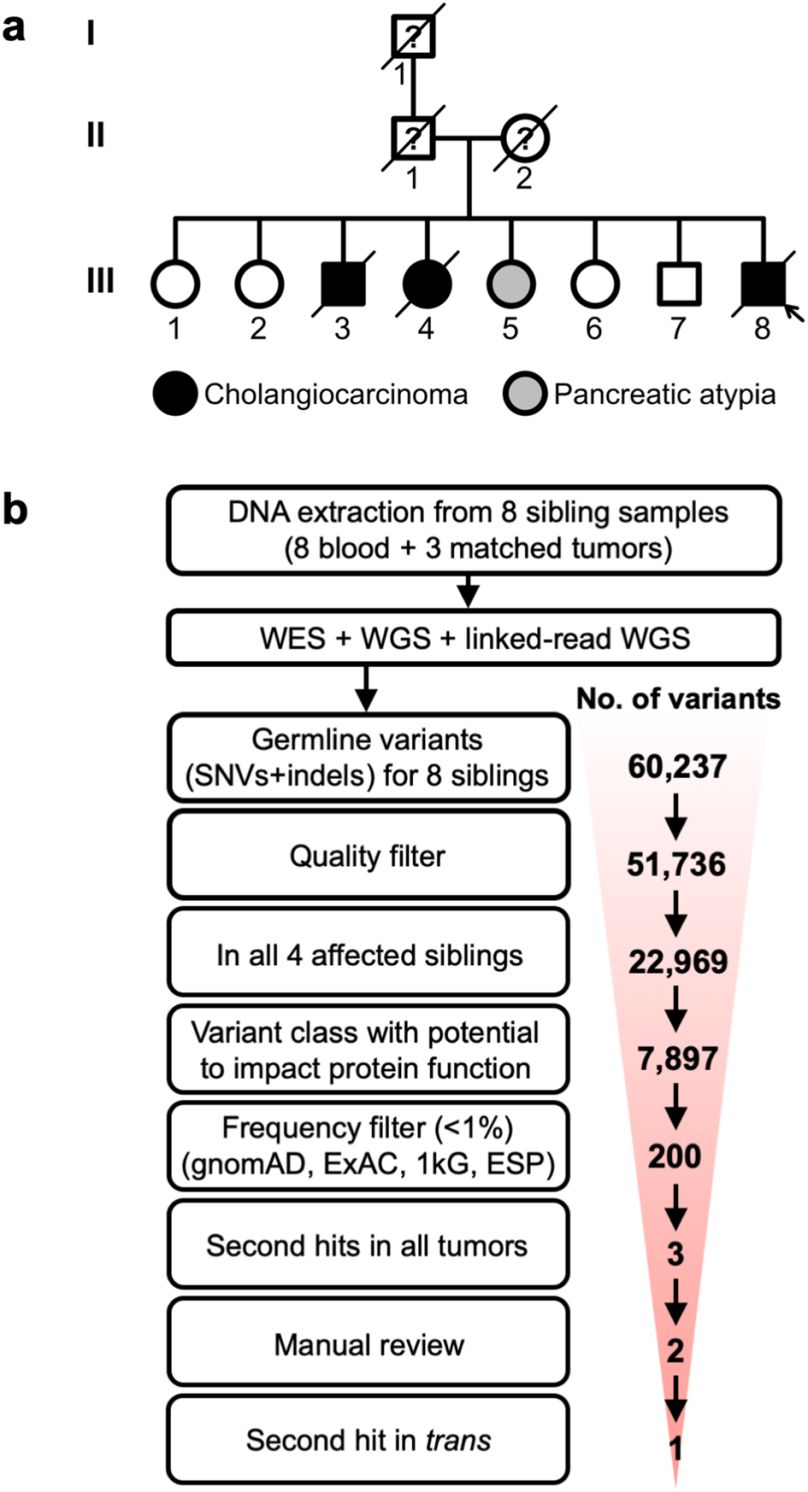
Identification of a putative germline predisposition variant in a family with inherited CCA. (**a**) This pedigree depicts a family with an inherited predisposition to biliary cancers. The current study had access to samples from generation III of the family. (**b**) The number of putative germline variants (single nucleotide variants and insertion-deletions) is indicated for each stage of filtering.

We identified a family with a high incidence of perihilar CCA (Supplementary Table S1 and Fig. 1a). As noted previously, perihilar CCA was present in three of eight siblings (III:3, III:4, III:8) in generation III of the family. The pathology review from the tumors taken at surgical resection and CT imaging studies confirmed this diagnosis among these siblings. One of the eight siblings was diagnosed with pancreatic atypia (III:5). The average age at diagnosis in generation III was approximately 60 years. In addition, gastrointestinal cancers affected all three generations of this family. Specifically, the parents and the paternal grandfather were reported to have cancers related to the GI tract. The father (II:1) was reported to have had pancreatic and prostate cancer at the time of death, and the mother (II:2) had pancreatic and cecal tumors. The family also reported that the paternal grandfather (I:1) was diagnosed with stomach cancer. We enrolled and collected blood from all siblings in generation III of the family, both affected and unaffected.

Supplementary Fig. S1 outlines our approach for identifying candidate genes and germline mutations that may account for the increased incidence of CCA in this family. We conducted both exome and whole genome analysis including studies with linked reads that provided haplotype information. Sequencing and alignment metrics are available in Supplementary Table S2 and phasing metrics are available in Supplementary Table S3. First, we examined the germline exome variants of the eight siblings. Overall, we identified on average 33,203 single nucleotide variants (SNVs) per sibling and 3,994 insertion-deletions (indels) per sibling (Supplementary Table S4). When we combined the variants of all individuals, there were a total of 46,133 and 5,603 high quality SNVs and indels, respectively, among the eight siblings.

To identify germline mutations associated with the affected family members, we used the following criteria: the germline variant was present in all of the affected siblings albeit it could be present in unaffected siblings as well, assuming incomplete penetrance; the variant had the potential to alter protein function (i.e. the variant class had to be one of: missense, nonsense, splice site, stop lost, frameshift, in-frame); the variant had to occur at low frequency (i.e. less than 1%) in the ‘healthy’ population. This germline filtering strategy is similar to those used in other WES studies to identify putative causal variants in Mendelian disorders^18^.

Approximately 44% (22,969) of the variants we identified were present in all four of the affected siblings (Supplementary Table S5 and Fig. 1b). Of the variants in the affected siblings, 34% (N = 7897) had the potential to impact protein function. For the SNVs, there were 6,610 nonsynonymous variants, 656 splice site variants, and 75 variants affecting stop codons (Supplementary Table S6). For the indels, there were 203 in-frame variants, 191 frameshift variants, and 162 splice site variants (Supplementary Table S6). We cross-validated the variants with the ESP, ExAC and gnomAD databases, which allowed us to eliminate population polymorphisms that were unlikely to be the cause of a rare hereditary condition^19^. In total, 43 SNVs and 157 indels remained as putative germline mutations, present in 148 genes (Supplementary Table S5 and Fig. 1b).

The germline analysis of this family did not identify mutations in any of the previously reported CCA and pancreaticobiliary cancer predisposition genes. Familial cases of CCA have been reported in Lynch syndrome (MIM: 120,435) caused by mutations in the DNA mismatch repair genes which include *MLH1*, *MSH2*, *MSH6*, *PMS1*, and *PMS2*^20^ and in BAP1 tumor predisposition syndrome (MIM: 614,327), where the *BAP1* tumor suppressor gene is mutated^21,22^. Genetic studies of hereditary pancreatic cancers have included CCA given the similarities of their embryonic tissue origins and epithelial features which involve secretory physiologic functions^7,23^. These genes include *BRCA1*^24^, *BRCA2*^25^, *PALB2*^26^, *ATM*^27^, *STK11*^28^, *CDKN2A*^29^, and *BRIP1*^30^. We did not find mutations in any of the above-mentioned genes or in genes with mutations reported as risk factors for pancreatic cancer from recent genome-wide association studies (*TNS3*, *NOC2L*, *HNF4G*, *HNF1B, GRP*, *UHMK1*, *AP1G2*, *DNTA*, *CHST6*, *FGFR3* and *EPHA1*)^27,31–33^. In addition, we used whole genome sequencing to identify germline deletions, rearrangements or other SVs that may be segregating with the affected individuals. We determined that there were no germline rearrangements associated with the affected individuals that implicated a susceptibility locus (Supplementary Table S7).

### Characterization of somatic mutations in CCA

We evaluated whether any of the 148 candidate genes identified in our germline analysis also harbored somatic alterations in the tumors of the three siblings with CCA, thus indicating putative biallelic genetic events. We conducted whole exome and whole genome sequencing on three tumor-normal sample pairs (patient III:3, III:4, III:8) to identify somatic cancer mutations and other genetic aberrations. We examined somatic SNVs and indels to identify putative inactivating mutations that could represent second hits (Tables S8–S11). We also examined genomic copy number changes as a way of ascertaining whether any deletions overlapped with our candidate germline mutations. The tumor of patient III:3 had five somatic copy number deletions with an average size of 49.8 Mb and containing a total of 3050 genes. In patient III:4, there were 32 somatic deletions with an average size of 34.8 Mb containing 9,620 genes. Patient III:8 had 20 somatically deleted regions with an average size of 24.9 Mb and containing 3,053 genes (Supplementary Table S12).

We identified three candidate germline variants in genes that also harbored somatic mutations among the affected individuals after filtering (Supplementary Table S13). The first was a missense mutation in *SPSB1* (NM_025106.4: c.945C>T p.Arg202Trp (p.R202W]) present in all eight siblings. The second was a nonsense mutation in *ATG7* (c.2000C>T [p.R659*]) present in four affected siblings as well as two unaffected siblings (III:2 and III:6). The third was a missense mutation in the *PP2D1* gene (NM_001252657.2: c.1898G>A p.Pro547Leu (p.P547L)) but it was eliminated as a candidate due to a lack of functional evidence based on variant impact predictions. The *PP2D1* substitution was scored as being benign and tolerant as per PolyPhen and SIFT analysis, respectively. Also, this mutation had a low CADD score (5.309) denoting low predicted pathogenicity (Supplementary Table S14). For *SPSB1* and *ATG7*, we identified both germline mutations and somatic alterations among all three affected individuals’ tumors (Tables S13 and S14). Specifically, all three patient tumor samples harbored somatic copy number deletions that included these two genes (Tables S12 and S13).

### Linked read sequencing confirmed second hit for *ATG7*

**Figure 2:**
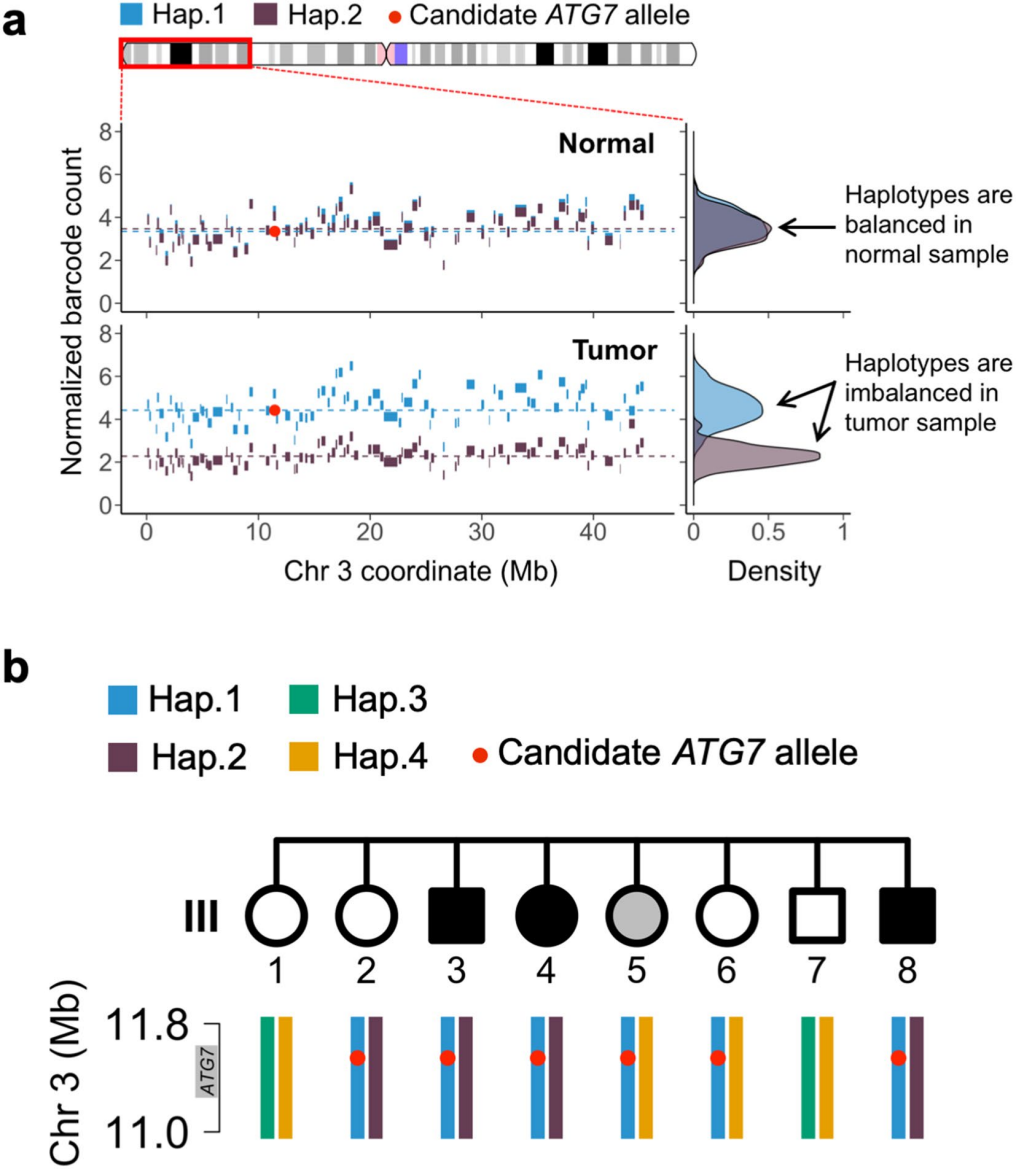
Haplotype analysis of the candidate *ATG7* allele (p.R659*). (**a**) Extended haplotype of the 45 Mb deleted region of chromosome 3p in individual III:8. The blocks indicate the original fragmented haplotypes, and their color denotes their subsequent assignment to haplotypes covering many Megabases. The candidate *ATG7* allele exists in haplotype 1 (blue), which was the non-deleted haplotype in the tumor of this individual. (**b**) Germline haplotype analysis of siblings for the *ATG7* genomic region. The haplotype segregation across all eight siblings in generation III was determined for the 0.8 Mb genomic region surrounding the *ATG7* allele (red dot). The candidate *ATG7* allele exists in haplotype 1 (blue).

Next, we determined whether genomic deletions of the two candidate genes led to the loss of the remaining wildtype (WT) allele in the tumor sample. For this haplotype analysis, we applied linked-read sequencing to the tumor of patient III:8 to ascertain whether the somatic copy number deletion affected the mutant allele or the wildtype allele. We applied a chromosomal haplotyping method to link phase blocks^34^ and identified 14 chromosome arms where extended haplotypes covered anywhere from 1 to 98% of a given chromosome arm (Supplementary Table S15). *SPSB1* is located on chromosome arm 1p in an encompassing 36.6 Mb haplotype (p = 5.3 × 10^–13^). *ATG7* is located on chromosome arm 3p in an encompassing 44.4 Mb haplotype (p = 2.2 × 10^–16^) (Supplementary Table S15). To visualize the haplotype structure across these genomic deletions, we plotted the two haplotypes such that individual phase blocks are color-coded according to their assignment to an extended genomic haplotype (Figs. 2a and S2).

In the case of *SPSB1*, we discovered that the germline mutation (p.R202W) was deleted in the tumor genome and thus the remaining allele was wildtype (see Supplementary Table S16 and Supplementary Fig. S2 online). Based on the retention of the wildtype allele, we eliminated it as a possible tumor suppressor. Conversely, in the case of ATG7, we found that the wildtype *ATG7* allele was deleted in the tumor and thus the haplotype with the *ATG7* germline mutation (p.R659*) was retained, leading to loss of heterozygosity (LOH) in tumor (Supplementary Table S16 and Fig. 2a). Together, the germline mutation and genomic deletion identified an *ATG7* loss of function among the CCAs from the affected individuals. Using the extended haplotype information from the germline analysis of all generation III siblings, we confirmed that all affected individuals had inherited the extended haplotype with the candidate p.R659* allele in the *ATG7* genomic region (Fig. 2b).

### Identification of an *ATG7* polymorphism associated with CCA

We leveraged the Icelandic population for a study of *ATG7* association with CCA. We analyzed sequence variants in *ATG7* identified in whole genome sequence data from 8,453 Icelanders sequenced to a median depth of 32 × ^35^. Based on the SNVs and single nucleotide polymorphisms (SNPs) that were identified from the sequencing data, we imputed the *ATG7* genotypes of an additional 150,656 Icelanders with SNP array information. Additional familial imputation, using the nationwide Icelandic genealogical database, allowed these genotypes to be propagated into 294,212 un-genotyped close relatives of the chip-genotyped individuals. Thus, the overall sample size was adequately powered to detect the association of specific *ATG7* variants and polymorphisms with an increased risk of developing CCA.

Six coding variants in *ATG7* reached our threshold of imputation quality (Supplementary Table S17). Using information from the Icelandic Cancer Registry (ICR), we tested the association between these variants and affected individuals with CCA (*N* = 353). This analysis revealed an association between the minor allele of rs146589465 (rs146589465-G) and increased risk of CCA (OR 6.56, *P* = 1.3 × 10^–3^; Table 1). We assessed the quality of the imputation of rs146589465 by directly genotyping imputed carriers (*N* = 154) and non-carriers (*N* = 1180). The concordance between imputed and directly measured genotypes was 0.999 (Supplementary Table S18).

**Table 1:** Association analysis of rs146589465-G.

A1^a^	A2^a^	Freq. A1^a^ [%]	Phenotype	*N* cases	*N* controls	*P-*value	OR^a^ (A1)	95% CI
G	C	0.12	Cholangiocarcinoma	353	233,169	1.3 × 10^–3^	6.56	(2.085–20.64)
			Pituitary adenoma	422	303,642	3.9 × 10^–3^	5.22	(1.699–16.03)
			Hepatocellular carcinoma	280	301,578	2.9 × 10^–2^	4.95	(1.178–20.80)

Shown are associations for rs146589465-G in the Icelandic cohort that reached a threshold of *P*-value less than 5.0 × 10^–2^. The variant results in coding change p.D522E in ATG7.

^a^A1 and A2 stand for the two alleles tested for the marker. Odds-ratio (OR) and population allele frequency (Freq. A1) are provided for allele A1.

The variant rs146589465-G results in an aspartic acid to glutamic acid substitution at position 522 in the ATG7 protein (p.D522E). The variant is rare and is present in 1/400 individuals in the Icelandic population (allelic frequency is 0.12%). It is also present at a similar frequency in individuals of European ancestry per the gnomAD database (Supplementary Table S17). It is present only at a very low frequency in other populations, with its highest frequency being ~ 0.1% in the Latino population. To assess whether rs146589465 was associated with increased risk for other cancer types, we tested 19 additional tumor types using information from the ICR (Supplementary Table S19). The risk of hepatocellular carcinoma (OR = 4.95, *P* = 2.9 × 10^–2^; Table 1) and pituitary adenoma (*N* = 422; OR = 5.22, *P* = 3.9 × 10^–3^) was also elevated with rs146589465-G (Table 1).

**Figure 3:**
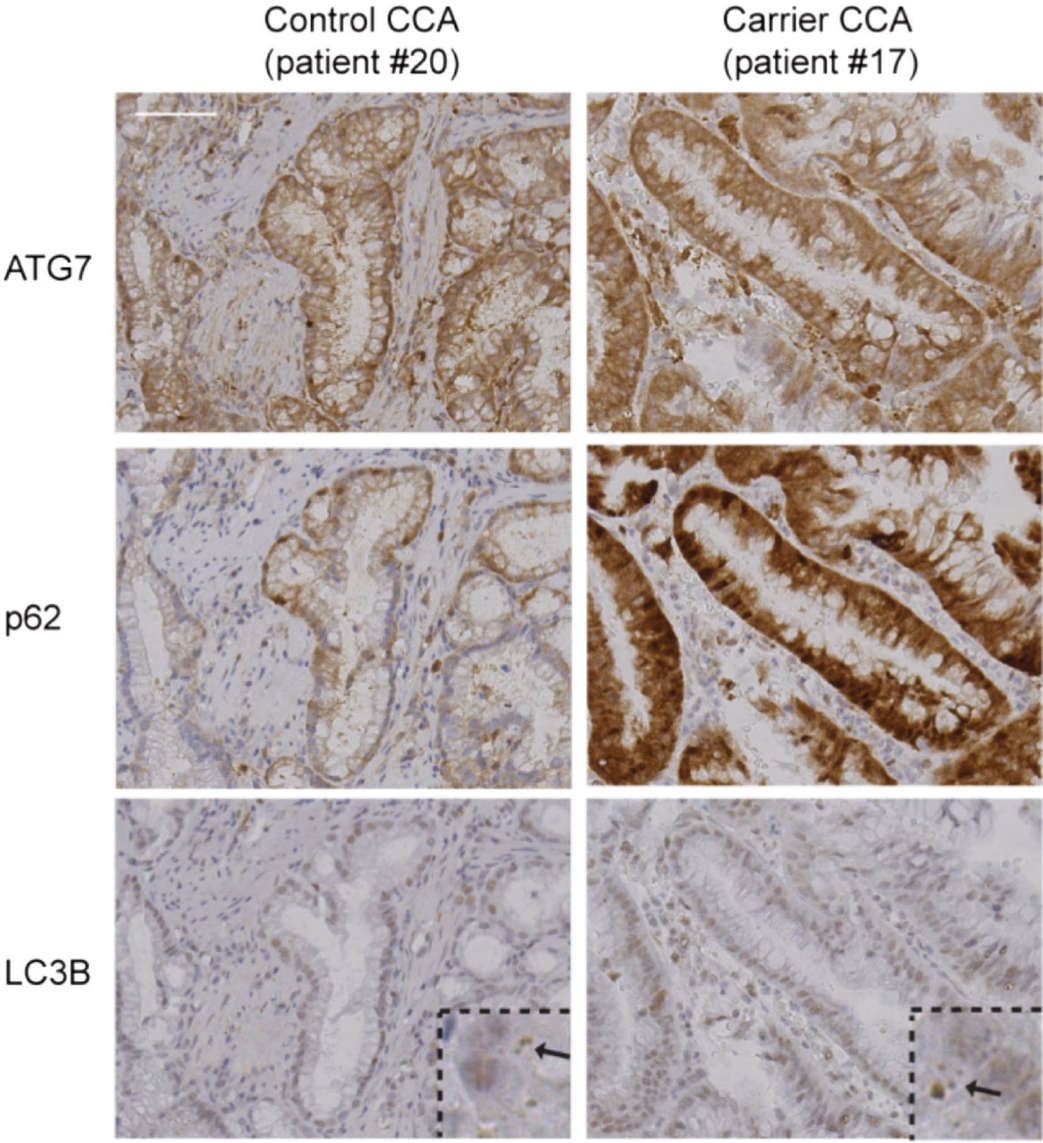
Increased p62 expression in tumors of ATG7 variant (p.D522E) carriers. The microscopic images show sections of CCA from an rs146589465 (p.D522E) carrier (patient #17) and a non-carrier control (patient #20) stained with antibodies against ATG7, p62 and LC3B. For the LC3B staining, a higher magnification window is shown and the arrows point to LC3 dots that are indicative of autophagosomes. Scale bar represents 20 µm and applies to all panels.

To evaluate the association of the ATG7 rs146589465-G (p.D522E) variant with autophagy defects in patient samples, we analyzed CCA as well as hepatocellular tumors from 6 carriers and 22 non-carriers (Tables S20 and S21, Figs. 3 and S3). There was no difference in the expression of ATG7 when comparing tumors from carriers and non-carriers, suggesting that the variant does not alter the expression of the ATG7 protein. In addition, there was no difference in expression of the autophagy marker LC3 nor in the pattern of LC3 staining in the tissue samples. Interestingly, we observed significantly higher levels of cytoplasmic p62 protein in tumor samples from rs146589465-G carriers compared to those of non-carriers (*P* = 0.003). As noted, the accumulation of p62 is frequently used as an indicator for autophagy defects since this protein is degraded during normal autophagy activity^36^. For example, p62 levels are increased in hepatomas developed from *Atg7* knockout mice^16^.

### Protein structure implicates ATG7 R659* as a putative loss-of-function mutation

**Figure 4:**
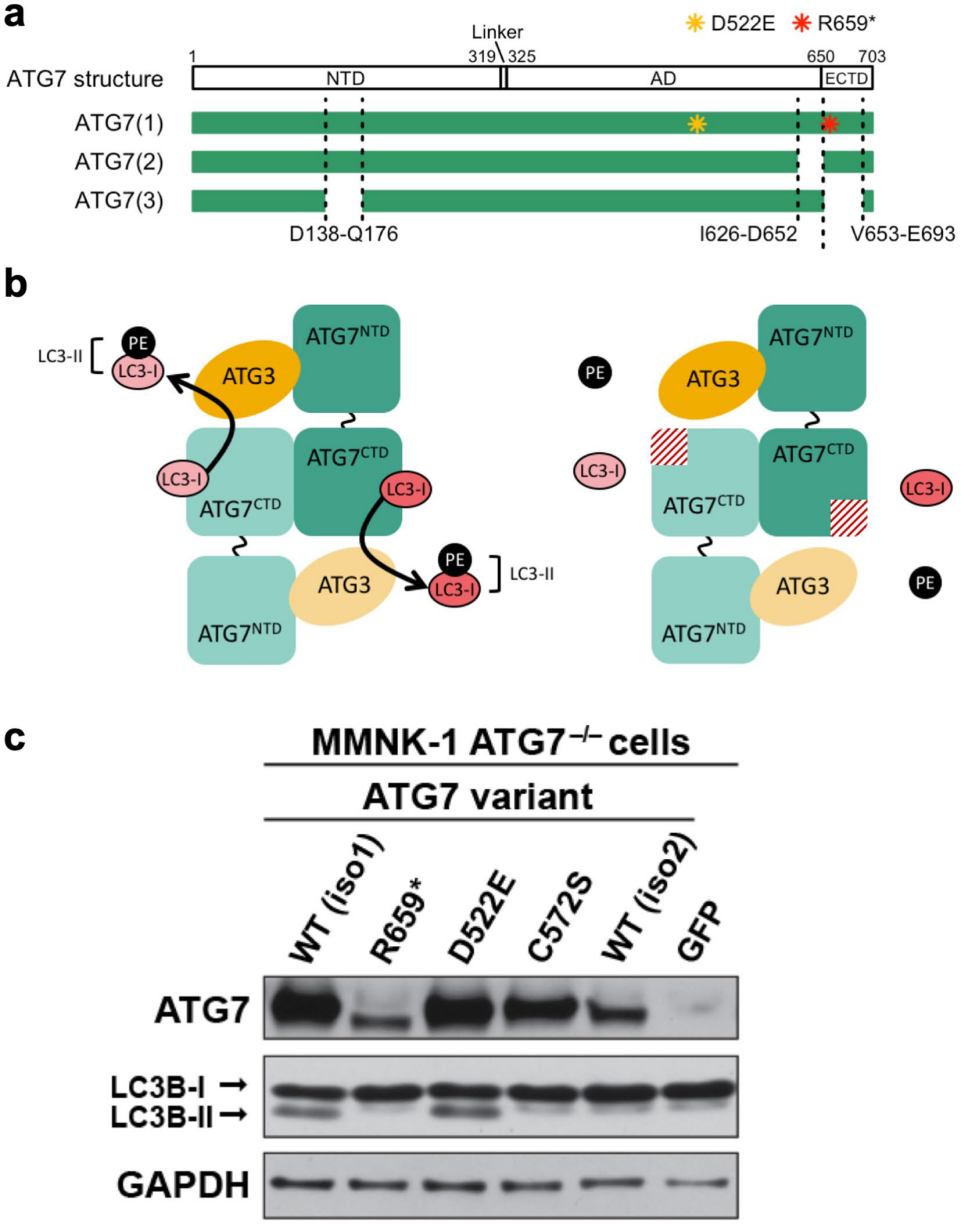
ATG7 p.R659* is a loss-of-function mutation. (**a**) The predicted structure of ATG7 contains an N-terminal domain (NTD), linker region, adenylation domain (AD), and extreme C-terminal domain (ECTD). The p.R659* (R659*) and p.D522E (D522E) mutations are indicated in isoform 1 of the ATG7 protein. (**b**) Predicted functional impact of ATG7 p.R659* mutation. Wildtype ATG7 and ATG3 conjugate ATG8 proteins such as LC3 with phosphatidylethanolamine (**PE**). We determined that the ATG7 R659* mutant lacks PE conjugation activity to LC3 as a result of the ECTD truncation. (**c**) Expression of ATG7 WT isoform 1 (iso1) and D522E restored the lipidation of LC3B whereas expression of ATG7 R659*, C572S, or WT isoform 2 (iso2) failed to lipidate LC3B from LC3B-I to LC3B-II. A Myc-tag was used to detect the expression of exogenous ATG7 from various vectors. A GFP expressing vector was used as a negative control. GAPDH was used as the loading control. Original blots are presented in Supplementary Fig. S9 online.

Using SWISS-MODEL we modeled and annotated the human ATG7 protein structure based on the yeast Atg7 protein (Figs. 4a and S4)^37^. Human ATG7 has E1-like activity similar to yeast Atg7^15^. The yeast Atg7 protein has been crystallized, revealing two conserved structural domains, namely the N-terminal domain (NTD) and the C-terminal domain (CTD)^38,39^. The NTD is responsible for Atg3 binding and the CTD is required for Atg8 (LC3/GABARAPs in mammals) recognition and binding. The C-terminal domain has two subdomains that include a homodimeric adenylation domain (AD) and an extreme C-terminal domain (ECTD). The human ATG7 p.R659 residue is in a highly conserved area of the ECTD and generates a stop codon leading to truncation of the ECTD (see Supplementary Fig. S4 online). Therefore, we speculate that ATG7 p.R659 is a loss-of-function mutation and will be unable to bind LC3. The human ATG7 p.D522 residue is located on the exposed exterior loop of the AD in a less conserved area, which may affect protein interactions (see Supplementary Fig. S4 online).

### Evaluating the impact of the *ATG7* germline mutation and polymorphism on autophagy

Human *ATG7* has three isoforms (Fig. 4A). Isoforms 1 and 2 are expressed in human tissues whereas isoform 3 is present at nearly undetectable levels^40^. ATG7 isoform 2 lacks a part of the region required for LC3 binding and is unable to lipidate LC3^40^. Thus, only isoform 1 of *ATG7* has the capacity to lipidate LC3 during autophagosome formation. In yeast, the ECTD is essential for Atg7 recognition and binding to Atg8. Along these lines, we conducted in vitro functional assays to determine whether the human *ATG7* variants identified in our study altered lipidation of LC3 (Fig. 4b)^38,39^. We hypothesized that the germline mutation from the family may have a more significant impact on specific autophagy functions than the population variant which was associated with a lower incidence of cholangiocarcinoma.

To study the functional impact of *ATG7* mutants, we engineered a human cholangiocyte cell line (MMNK-1) with an *ATG7* null background. The MMNK-1 cell line was derived from the human bile duct, thus representing the same primary tissue from which CCA arises. We applied CRISPR/Cas9 to generate a homozygous deletion in exon 2 of *ATG7* in MMNK-1 cells and isolated / expanded individual cells to screen for isogenic MMNK-1 *ATG7*^*-/-*^ clones. We confirmed the complete loss of ATG7 protein expression in MMNK-1 *ATG7*^*-/-*^ cells (see Supplementary Fig. S5 online). LC3B lipidation was evaluated by western blotting where PE-bound LC3B-II. The lipidated form migrates faster than the non-lipidated LC3B-I. There was a notable reduction of LC3B-II in MMNK-1 ATG7 CRISPR/Cas9 pools compared to the MMNK-1 cells, suggesting *ATG*7 was successfully knocked out in majority of cells. Meanwhile, the isogenic MMNK-1 *ATG7*^*-/-*^ cells derived from a single clone lacked a lower LC3B-II band, demonstrating complete loss of ATG7 function (see Supplementary Fig. S5 online). The presence of the CRISPR-mediated homozygous deletion was confirmed with Sanger sequencing and targeted amplicon sequencing (see Supplementary Fig. S6 online).

To evaluate the function of the ATG7 variants, isogenic MMNK-1 ATG7^-/-^ cells were transfected with vectors expressing WT ATG7 isoform 1, p.R659* (familial germline mutation), p.D522E (the Icelandic population variant), p.C572S (a known inactivating mutation of ATG7), and wildtype ATG7 isoform 2 (Methods). The expression level of ATG7 R659* was notably lower than the wildtype protein (Fig. 4c) despite similar levels of plasmid input and mRNA expression (see Supplementary Fig. S7 online). This result pointed to the truncating mutation impacting the stability of the ATG7 protein. In comparison, the ATG7 D522E protein was expressed at a similar level to the wildtype.

Subsequently, we assessed the activity of ATG7 mutants by the level of lipidated LC3B. Transient expression of either wildtype ATG7 isoform 1 or D522E enabled conversion of LC3B-I to LC3B-II in MMNK-1 ATG7^-/-^ cells. In comparison, expression of R659*, C572S, or the ATG7 isoform 2 failed to convert LC3B-I to LC3B-II (Fig. 4c). Therefore, the R659* mutation resulted in a loss-of-function of the ATG7 lipidation activity.

To conduct long term functional assays of the *ATG7* variants and their impact on cholangiocytes, we established stable MMNK-1 ATG7^−/−^ cell lines re-expressing ATG7 WT and mutants by lentiviral transduction. There was substantially lower expression of ATG7 protein in cell lines expressing the R659* mutation (see Supplementary Fig. S8 online), which confirmed our previous observation from transient transfection in Fig. 4c.

### ATG7 R659* impairs autophagy by inhibiting the formation of the ATG5-12 complex

**Figure 5:**
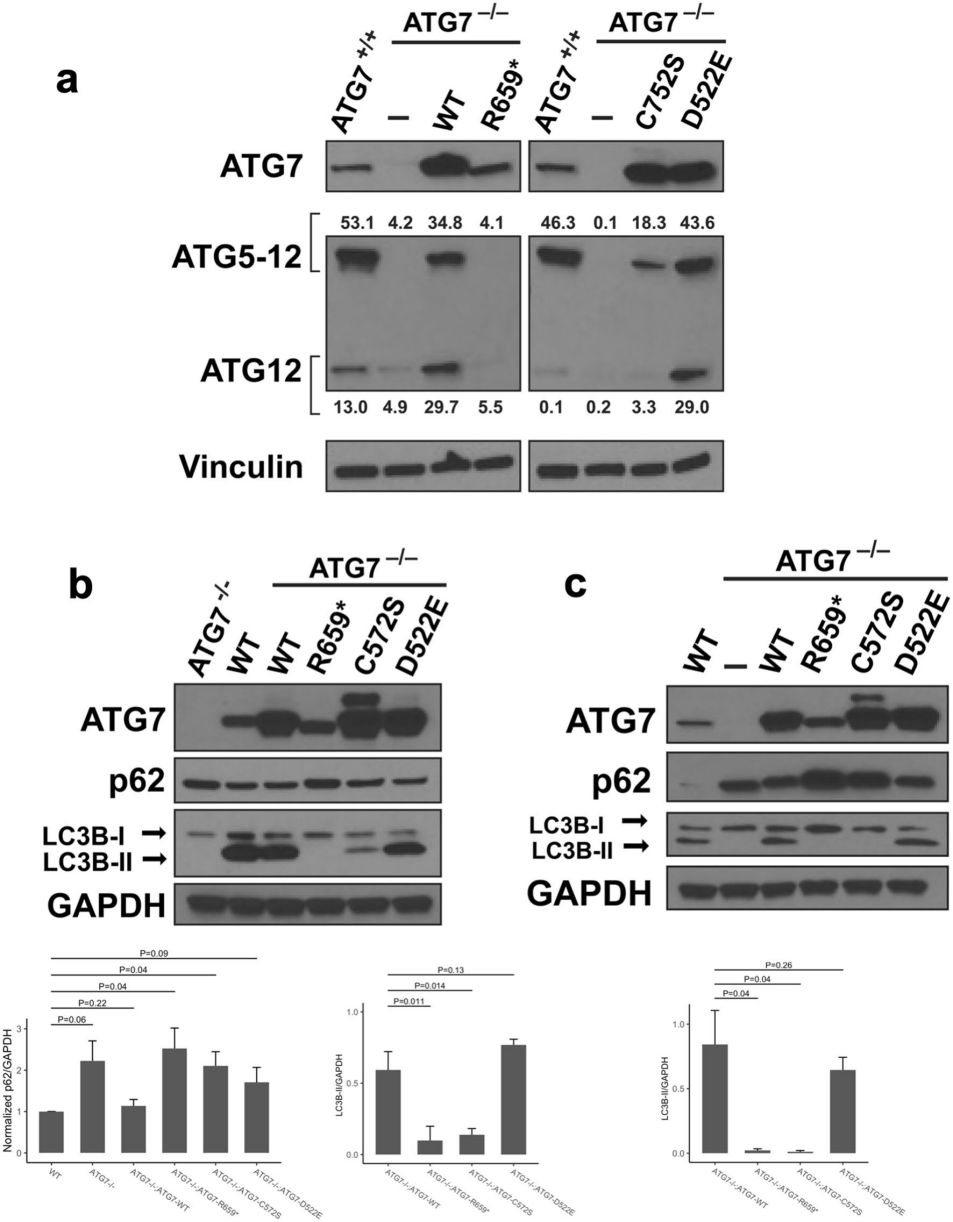
Impaired ATG5-12 complex formation and elevated p62 levels in cholangiocytes expressing ATG7 R659*. (**a**) Cholangiocytes expressing ATG7 R659* unable to form ATG5-12 complex. The ATG7 R659* and C572S variants fail to rescue the ATG5-12 complex formation defect observed in MMNK-1 ATG7^-/-^ cholangiocytes. Protein lysates were obtained from cell lines under basal conditions. Free ATG12 and the ATG5-12 complex can be detected at 15 kDa and 55 kDa, respectively, on the western blot. (**b**) Top: Elevated p62 expression due to autophagy defects in cholangiocytes expressing ATG7 mutants. Cholangiocytes expressing ATG7 WT, R659*, D522E, and C572S were cultured using normal growth media. Expression of ATG7, p62, LC3B, and GAPDH were detected on the western blot. Bottom left: Bar plot with y-axis showing normalized p62/GAPDH ratio and x-axis showing the distinct cell line genotypes. Normalized refers to the normalization of all genotypes in relation to the wild-type cell line. P-values are shown for statistical t-test comparisons. Bottom right: Bar plot with y-axis showing LC3B-II/GAPDH ratio and x-axis showing the distinct cell line genotypes. (**c**) Top: Cholangiocytes expressing ATG7 WT, R659*, D522E, and C572S starved with HBSS for 3 h. Expression of ATG7, p62, LC3B, and GAPDH were detected on the western blot. Bottom: Bar plot with y-axis showing LC3B-II/GAPDH ratio and x-axis showing the distinct cell line genotypes. Original blots are presented in Supplementary Fig. S9 online. Quantifications reflect the mean of triplicates experiments for conditions and genotypes. P values are provided from t-tests performed.

ATG7 is required for two conjugation cascades that result in the formation of the ATG5-12 complex and the subsequent lipidation of LC3/GABARAPs^15,41,42^. Our western blot studies showed that stable expression of the R659* familial germline variant in an MMNK-1 ATG7^-/-^ background failed to rescue the lipidation phenotype (see Supplementary Fig. S7 online). Therefore, we sought to determine whether the LC3 lipidation defect was the result of impaired ATG5-12 formation via western blotting. We assayed ATG12 using our stable cell line constructs in an ATG7^-/-^ background^43^. Our results showed defects in the formation of the ATG5-12 complex in ATG7 null, R659*- and C572S-expressing cell lines (Fig. 5a). Meanwhile, no abnormalities were noted for the ATG7 WT and D522E variant when compared to parental MMNK-1 ATG7^+/+^ cells. Similarly, we observed autophagy disruption by performing western blots against LC3B. When compared to Atg7 wild-type rescue cells, a null rescue using R569* or C572S resulted in impaired lipidation of LC3B-I to LC3B-II during basal and nutrient starvation conditions (Fig. 5b,c). No significant defect in LC3B-II lipidation was noted when D522E was used to rescue the Atg7-null cell line. Altogether, our results support a loss of function phenotype for the R659* germline variant during autophagy.

### ATG7 variants lead to p62 accumulation

To evaluate the autophagy response under metabolic stress, we assessed the level of ubiquitin-binding protein p62 in cholangiocytes expressing *ATG7* WT and mutants. The p62 protein is a key component for autophagic degradation of ubiquitinated proteins. Thus, p62 links the autophagy pathway and the ubiquitin–proteasome system for protein degradation^44^. We observed increased p62 levels in cholangiocytes expressing ATG7 R659* and C572S, and to a lesser extent, D522E during basal and starvation conditions (Fig. 5b,c). In addition, we observed that accumulation of p62 correlated with autophagy defects as evaluated by the impaired generation of LC3B-II in the null, R569* and C572S genotypes under basal and starvation conditions (Fig. 5b,c). These results involved a comparison to the wild-type and WT ATG7, under basal conditions and metabolic stress under starvation conditions. Our data demonstrated that expression of cancer associated ATG7 variants resulted in p62 accumulation in cholangiocyte cells under metabolic stress paralleling a tumorigenic environment.

## Discussion

In this study, we identified a novel germline mutation p.R659* and a rare variant p.D522E in *ATG7* associated with CCA, based on a comprehensive genetic analysis of an extended pedigree of familial CCA and a population genetic association study, respectively. Our study of the CCA pedigree leveraged the analysis of haplotypes shared among affected individuals in a family with CCA. We directly demonstrated that a somatic deletion of the *ATG7* wildtype allele in an affected tumor sample represented a second hit event.

*ATG7* plays an essential role in the autophagy pathway during autophagosome formation. Meanwhile, the role of autophagy in tumorigenesis is complicated, context-dependent and tissue-dependent^45^. Autophagy has been reported as tumor suppressive in some studies whereby autophagy deficiency led to tissue damage and genome instability. For example, heterozygous deletions of *BECN1 (Atg6)*, a member of the ATG gene family involved in autophagosome initiation, have been reported in human prostate, breast, and ovarian cancers, and *Becn1* deficiency led to spontaneous tumor development in mice^46,47^. Liver-specific autophagy deficiency due to Atg7 deletion led to the development of liver tumors in mice^17^. In other studies, autophagy activity promoted tumor growth by suppressing metabolic stress and supporting cancer cell survival. For example, autophagy was required for the development of KRAS-driven lung adenocarcinomas in mice^48^. Previous studies have linked germline autophagy gene mutations to a variety of hereditary disorders. For example, inherited mutations in *ATG5* have been implicated in childhood ataxia, a neurodegenerative condition where balance and coordination are lost as well as leading to developmental delay^49^. Specific polymorphisms have been associated with an elevated risk of certain autoimmune conditions. Notably, *ATG5* and *ATG16L2* polymorphisms have been associated with systemic lupus erythematosus^50,51^, and *ATG16L1* polymorphisms have been linked to Crohn’s disease^52,53^. However, the role of *ATG* family genes in cholangiocarcinoma remains unknown. Our study reports a germline mutation in *ATG7* strongly associated with CCA.

To study the functional impact of the ATG7 variants in the context of cholangiocytes, we established a human *ATG7* null bile duct cell line. Our studies evaluating LC3 lipidation and Atg5-12 complex formation demonstrated that the germline mutation ATG7 p.R659* is a loss-of-function mutation during autophagy. We found that deficiency in autophagy due to ATG7 p.R659* resulted in the accumulation of p62 in cholangiocytes. This was notable under metabolic stress. Studies have demonstrated that accumulation of p62 results in the activation of antioxidant response, coupled with c-Myc and mTORC1 activation that results in metabolic reprogramming beneficial to cancer cells^54,55^.

The rare variant p.D522E identified from a population genetic association analysis did not display a noticeable effect on ATG7 function, and ATG7 p.D522E functioned like the wildtype protein with regard to LC3 lipidation and the conjugation cascades. This finding was not surprising because the population variant p.D522E resulted in a more modest change from aspartic acid to glutamic acid in a less conserved region compared to the p.R659* mutation. However, p62 levels were elevated in the tumors of p.D522E carriers compared with control samples. The p62 protein is degraded by autophagy and thus its accumulation indicates impaired autophagy activity^56^. For example, notable p62 accumulation was observed in hepatocellular adenomas derived from liver-specific *Atg7* knockout mice^16,57^. Our findings suggest that the phenotypic effects of the p.D522E variant have less of an impact on autophagy. Therefore, the consequences of this polymorphism may be related to cumulative effects over a longer period of time, which could be seen in the tumors but is not detected in the cell culture model. In addition, the impact of p.D522E may occur through non-canonical autophagy pathways that are independent of LC3 lipidation or at later stages of canonical autophagy following lipidation. Further studies are needed to identify the precise cellular mechanisms affected by the p.D522E mutation.

In addition to its key role in autophagy, ATG7 has been shown to bind p53 directly and regulate subsequent transcription of the critical cell-cycle inhibitor *CDKN1A*, which encodes the p21 protein^58^. Mouse embryonic stem cells deficient in Atg7 fail to undergo cell cycle arrest, a defect not seen in Atg5- or Atg6-deficient embryos. The ability to directly bind to p53 and regulate cell cycle control is one potential mechanism by which the absence of ATG7 could lead to the initiation of tumorigenesis. Further studies are needed to examine the effect of mutant *ATG7* on levels of reactive oxygen species (ROS) and DNA damage, and also to determine how increased levels of p62 may be linked to cholangiocarcinoma development through the p62-KEAP1-NRF2 axis. In autophagy-deficient mouse livers, p62 accumulation leads to the upregulation of cytoprotective genes resulting in toxicity and liver damage^59^. In future studies, we will investigate the precise mechanism of how *ATG7* loss-of-function contributes to tumorigenesis in cholangiocarcinoma.

## Materials and methods

### Clinical samples

Our familial genetic study was conducted in compliance with the Helsinki Declaration, and the Institutional Review Board at Stanford University School of Medicine approved the study protocol (76,274). Samples were obtained with informed consent from the participants. Blood samples were obtained from eight siblings. We obtained matched tumor samples from the individuals affected with CCA (Supplementary Table S1 and Fig. 1). Genomic DNA extraction was performed using the Promega (Madison, WI) Maxwell 16 Blood DNA Purification Kit and 16 FFPE Plus LEV DNA Purification Kit for the germline blood samples and archival tumor samples, respectively.

### Exome sequencing and germline variant calling

We prepared libraries with the KAPA Hyper Prep kit (Kapa Biosystems, Wilmington, MA) and exome capture was performed with the Nextera Rapid Capture Exome kit (Illumina, San Diego, CA). We sequenced the libraries on either the Illumina HiSeq 2500 system with 100 by 100-bp paired-end reads or the Illumina NextSeq system with 150 by 150-bp paired-end reads. The resulting sequence reads were aligned to human genome build GRCh37.1 with the BWA-MEM algorithm of the Burrows-Wheeler Aligner (BWA) v0.7.4^60^. WES and alignment metrics are available in Supplementary Table S2.

We used the Sentieon variant caller^61^ (Mountain View, CA) v201711 to implement the GATK tools, following the GATK Best Practices for variant detection^62^. We generated a genome variant call format file for each sample using Sentieon Haplotyper and then performed joint variant calling on all eight samples with Sentieon GVCFtyper. We followed the GATK best practice recommendations for detecting SNVs and indels. Subsequently, we filtered out low quality variants without a ‘PASS’ filter. The remaining variants were annotated with the following: Variant Effect Predictor v74^63^ to assign general information including gene, amino acid, and mutation class; CADD v1.3^64^ to generate a pathogenicity score for each variant; and ESP, ExAC and gnomAD^65^ to determine the population allele frequency of each variant.

### Genome sequencing and haplotyping

To determine haplotypes over extended genomic segments (i.e. Megabases) we used linked read whole genome sequencing. This sequencing approach enables one to use barcoded short reads to obtain long-range genomic information from high molecular weight DNA^66^. With this long-range data, we performed germline SV detection and characterized the extended haplotypes across the entire genome for all eight siblings. For all of the pedigree germline samples, we prepared sequencing libraries with the Chromium Gel Bead and Library Kit and the Chromium Instrument (10X Genomics, Pleasanton, CA). These libraries were sequenced on the Illumina HiSeq X system with 150 by 150-bp paired-end reads. The resulting BCL files were converted to fastq files using Long Ranger (v2.1.2) ‘mkfastq’, then Long Ranger ‘wgs’ was run to align the reads to GRCh37.1, detect and phase SNVs/indels, and detect SVs. Linked read sequencing and alignment metrics are available in Supplementary Table S2, and phasing-haplotype metrics are available in Supplementary Table S3.

For the detection of somatic SNVs and indels, we performed high depth WES on three CCA samples in combination with the results from the matched normal blood samples in the germline analysis (above). Sequencing libraries for two of the tumor samples (patient III:3 and III:8) were generated using the KAPA Hyper Prep kit (Kapa Biosystems). The other tumor sample (patient III:4) had lower quality DNA and was prepared using the GemCode Library Kit (10X Genomics) to retain long-range information for improved read alignment. Exome capture was performed with the Nextera Rapid Capture Exome kit (Illumina). We sequenced the libraries on the Illumina HiSeq system with 100 by 100-bp paired-end reads or the Illumina NextSeq system with 150 by 150-bp paired-end reads. The sequence reads were aligned to human genome build GRCh37.1 with the BWA-MEM algorithm of BWA v0.7.4^60^. For the GemCode library, the BCL files were converted to fastq files using Long Ranger (v2.1.2) ‘mkfastq’, then Long Ranger (v2.1.2) ‘wgs’ was run to align the reads to GRCh37.1.

The mean on-target coverage for the tumor WES data ranged from 88 to 178×; sequencing and alignment metrics are available in Supplementary Table S2. We used the Sentieon v201711 TNsnv tool (similar to the MuTect algorithm) and TNhaplotyper tool (similar to the MuTect2 algorithm) to detect somatic SNVs and small indels using the WES data of each tumor/normal pair and retained only those variants with a ‘PASS’ filter. We annotated the variants with the same methodology as in the ‘Germline analysis’ (above), and also applied the same filters: the variant had to have the potential to affect protein function and the variant had to occur at low frequency (i.e. less than 1%) in population frequency data. For patient III:4, we used the WES data to identify CNVs using the CNVkit tool and determined copy number changes^67^.

We performed WGS on tumor-normal pairs from patients III:3 and III:8. Sequencing libraries were generated using the KAPA Hyper Prep kit (Kapa Biosystems). The libraries for patient III:3 were sequenced on the Illumina HiSeq 4000 system with 150 by 150-bp paired-end reads and the libraries for patient III:8 were sequenced on the Illumina HiSeq X system with 150 by 150-bp paired-end reads. The resulting sequence reads were aligned to human genome build GRCh37.1 with the BWA-MEM algorithm of BWA v0.7.4^60^. WGS and alignment metrics are available in Supplementary Table S2. To identify candidate genes where the second (i.e. ‘wildtype’) allele could have been lost due a genomic deletion or gene conversion, we ran the BICseq2^68^ CNV detection tool on these tumor-normal pairs.

We used linked read WGS to retain long-range genomic information from one of the tumor samples (patient III:8). We prepared the sequencing library for the tumor sample using the Chromium Library Kit (10X Genomics). The library was sequenced on the Illumina HiSeq X system with 150 by 150-bp paired-end reads. The resulting BCL files were converted to fastq files using Long Ranger (v2.1.2) ‘mkfastq’, then Long Ranger (v2.1.2) ‘wgs’ was run to align the reads to GRCh37.1, detect and phase SNVs/indels, and detect SVs.

We determined whether each candidate causal mutation was in *cis* or in *trans* with somatic deletion events in the tumor. For this haplotype analysis, we used linked read data to generate digital karyotypes as described in Bell et al*.*^34^. This method generates extended haplotypes from Mb segments, by combining adjacent phase blocks extrapolated from allelic imbalances. The Mb haplotypes are quantitatively determined from the differences in the linked read barcode counts among haplotypes. Using this process, we determined the alleles that belonged to each haplotype in regions with genomic deletions.

### Population genetic study of CCA

Individuals affected with CCA were identified through the ICR and samples were obtained with informed consent from the participants. All sample identifiers were encrypted in accordance with the regulations of the Icelandic Data Protection Authority. Approval for the study was granted by the Icelandic National Bioethics Committee (ref. 00/097) and the Icelandic Data Protection Authority (ref. 2001020223).

We identified all of the known variants occurring in *ATG7* for the Icelandic population as previously described^35^. A total of 31.6 million SNVs and short indels that met quality control criteria were identified in the genomes of 8453 sequenced Icelanders. These variants were then imputed into 150,656 Icelanders genotyped with Illumina SNP chips, and their genotypes were phased using long-range phasing^69,70^. To increase the sample size and power for genetic association analysis, we used genealogical deduction of carrier status for 294,212 relatives lacking array-based genotypes. Subsequently, *ATG7* variant association testing for case–control analysis was performed using logistic regression. The quality of the imputation was evaluated by comparing imputed genotypes to genotypes obtained by direct genotyping. Individual *ATG7* genotyping was performed by applying the Centaurus (Nanogen) platform.

### Tumor immunohistochemistry

Immunohistochemistry was performed on 3 μm sections from paraffin-embedded tumors. Following deparaffinization, samples were rehydrated and subjected to heat-induced epitope retrieval. Tris/EDTA buffer pH 9 was used for the ATG7 (clone EP1759Y, Millipore (Burlington, MA) 04–1055; 1:1000), LC3B (clone D11, Cell Signaling Technology (Danvers, MA) 3868; 1:250) and p62 (Enzo (New York, NY) PW9860; 1:100) antibodies in a 98.2 °C water bath (5/20/20). Endogenous peroxidase activity was blocked with 3% hydrogen peroxide. After incubation with the respective primary antibodies for 30 min at room temperature, EnVision FLEX Kit (DAKO, Glostrup, Denmark) was used for detection. Microscope images of stained sections were scored in a blinded manner. A chi-square test was performed to assess whether staining patterns significantly differed between carrier and control samples.

### Cell lines

We used both human and mouse cell lines for these experiments. The immortalized human cholangiocyte cell line MMNK-1, and mouse embryonic fibroblasts (MEF) *Atg7*^*-/-*^ and MEF *Atg7*^+*/*+^, were obtained from RIKEN BioResource Center Cell Bank (Wako, Saitama, Japan). Cells were maintained in DMEM supplemented with 10% fetal bovine serum at 37 °C and 5% CO_2_. For characterizing these cells, we extracted genomic DNA and total RNA using the Maxwell 16 Cell LEV DNA Purification Kit and the Maxwell 16 Cell LEV Total RNA Purification Kit (Promega), respectively.

### Generation, screening, and genotyping of isogenic MMNK-1 ATG7^-/-^ cell lines

Using CRISPR-Cas9, exon 2 of *ATG7* was targeted for knockout in the MMNK-1 cell population (ZeClinics, Barcelona, Spain). Guide RNAs targeting exon 2 of *ATG7* were cloned into the PX458 and PX459 vectors. The guide RNA sequences are listed below.

*ATG7* sgRNA22_Top: 5ʹ- CACCGAACTGCAGTTTAGAGAGTCC -3ʹ.

*ATG7* sgRNA22_Bottom: 5ʹ- AAACGGACTCTCTAAACTGCAGTTC -3ʹ.

*ATG7* sgRNA119_Top: 5ʹ- CACCGAAGCTGAACGAGTATCGGC -3ʹ.

*ATG7* sgRNA119_Bottom: 5ʹ- AAACGCCGATACTCGTTCAGCTTC -3ʹ.

MMNK-1 cells were transfected with both sgRNA vectors and then selected with puromycin. We sorted single MMNK-1 cells into 96 wells to generate isogenic MMNK-1 *ATG7*^-/-^ cell clones.

To screen for isogenic MMNK-1 *ATG7*^-/-^ clones, we examined the expression of ATG7 protein using western blotting and selected the individual clones with complete loss of ATG7 expression for further expansion.

To genotype the MMNK-1 *ATG7*^-/-^ clones and confirm the presence of deletion in the exon 2 of *ATG7*, we applied both sanger sequencing and Illumina short read sequencing to sequence the area targeted by CRISPR/Cas9 knockout following the standard procedure as described previously^71^. The primers used to generate the PCR amplicon for sequencing are list below:

ATG7_exon_F: GTTGTGTTTCAAGGTAGCCTGT.

ATG7_exon_R: TCTCTTCCCACAGCAATGCT.

### Human *ATG7* variant plasmids for transient transfection experiments

The plasmid pCMV-myc-Atg7(2) expressing *ATG7* isoform 2 was a gift from Toren Finkel (Addgene plasmid #24921)^72^. The plasmid pCMV-myc-Atg7(1) expressing ATG7 isoform 1 was derived from pCMV-myc-Atg7(2) using the Q5 site-directed mutagenesis kit (NEB, Ipswich, MA). Subsequently, we used pCMV-myc-Atg7(1) to introduce the *ATG7* mutations p.R659*, p.D522E and p.C572S using the Q5 site-directed mutagenesis kit. After subcloning, all of the *ATG7* mutations in the vectors were confirmed with Sanger sequencing. The pCMV3-C-GFPSpark vector was used as the control (Sino Biological, Beijing, China). The primers are listed below:

*ATG7* p.R659*_F: 5ʹ- GTTCTTGATCAATATGAATGAGAAGGATTTAAC -3ʹ.

*ATG7* p.R659*_R: 5ʹ- TTTGGAAGAACAAGCTGTACATTTG -3ʹ.

*ATG7* p.D522E_F: 5ʹ- AGCTGGGGAGTTGTGTCCA -3ʹ.

*ATG7* p.D522E_R: 5ʹ- CCTTGCTGCTTTGGTTTCTTCA -3ʹ.

*ATG7* p.C572S_F: 5ʹ- CTTGGACCAGCAGTCCACTGTGAGTCG -3ʹ.

*ATG7* p.C572S_R: 5ʹ- GTCCGGTCTCTGGTTGAATCTCCTGG -3ʹ.

### Transient transfection of *ATG7* with mutations

Human MMNK-1 *ATG7*^-/-^ or mouse MEF *Atg7*^*-/-*^ cells were transfected with 2.5 μg of plasmid expressing WT ATG7 (isoform 1), ATG7 p.R659*, ATG7 p.D522E, ATG7 p.C572S or GFP control, using Lipofectamine 2000. Cells were starved in EBSS for 3 h and harvested for protein extraction 24 h post-transfection.

### Western blotting

Cell lysates (20 μg) were separated on 4–20% precast polyacrylamide gels (Bio-Rad, Hercules, CA) and were transferred to nitrocellulose membranes. The following antibodies were used for immunoblotting: ATG7 (D12B11, Cell Signaling), ATG12 (D88H11, Cell Signaling), LC3B (D11, Cell Signaling), p62/SQSTM1 (D-3, Santa Cruz Biotechnology (Dallas, TX)), Myc-Tag (9B11, Cell Signaling), Vinculin (E1E9V, Cell Signaling), and GAPDH (14C10, Cell Signaling).

For quantifications, each western blot experiment was performed as three independent biological replicates for all conditions and genotypes. Western blot films were exported to Adobe Photoshop CS3 and the images were inverted to black and white followed by inversion of the image for band measurements. The box tool was used to obtain the mean gray value of the film background and each individual band. Each western blot band was normalized to a loading internal control for quantification (e.g. GAPDH). Student's *t*-test statistical analysis was used to compare all genotypes and conditions tested using the mean from triplicate measurements.

### Quantification of *ATG7* plasmid input and gene expression

We determined the amount of plasmid maintained in the cell lines after transient transfection using droplet digital PCR (ddPCR). The ddPCR was performed on the Bio-Rad QX200 ddPCR system following the manufacturer instructions. Briefly, each 22µL ddPCR reaction used 1X EvaGreen ddPCR super-mix, 100 nM of primers and 10^–5^ ng of plasmid. The PCR master mix was partitioned and plated per Bio-Rad’s QX200 droplet generation protocol. After thermal cycling, the plate was transferred and read by the Bio-Rad QX200 Droplet Reader. Results were analyzed with the Quantasoft program (Bio-Rad).

The expression of mRNA transcripts from the cell lines was determined using a QuantStudio 6 Flex Real-Time PCR System (Thermo Fisher Scientific, Waltham, MA) with SYBR Green master mixes (Bio-Rad) following the manufacturer instructions. The *RPP30* gene was used as a housekeeping control. The primers used for *ATG7* ddPCR and cDNA amplification are listed below:

ATG7_F: 5ʹ-AAGCCATGATGTCGTCTTCC-3ʹ.

ATG7_R: 5ʹ- TCCTTGCTGCTTTGGTTTCT-3ʹ.

### Lentiviral constructs of the human *ATG7* variants

Constructs were generated using the BP/LR Gateway cloning system (Invitrogen, Cambridge, UK). Briefly, the *ATG7* constructs described above (WT, R659*, D522E, and C572S) were cloned from pENTR21 into the final vector pLenti-CMV-Blast-Destiny. After antibiotic selection, single colonies were used to inoculate liquid LB for subsequent use in plasmid extraction. The *ATG7* variants generated above were used to produce lentiviruses, which were subsequently transduced into MMNK-1 ATG7^-/-^ cells. Briefly, the lentiviral vectors were co-transfected with plasmids psPAX2 and pMD2.G into HEK293 cells at ~ 80–90% confluency. The lentivirus particles were harvested from cell media 48 h post-transfection and precipitated using PEG-it virus Precipitation Solution (Abcam, Cambridge, UK). Transduced MMNK-1 *ATG7*^-/-^ cells were selected by blasticidin.

## Supplementary Information

Supplementary Tables.

Supplementary Figures.

## Data Availability

All sequence data for this study has been submitted to dbGaP as fastq or bam files at the accession phs001593.v1.p1.
