# Supplementary material for: Germline variants of ATG7 in familial cholangiocarcinoma alter autophagy and p62
Source: Sci Rep. 2022 Jun 20;12:10333. doi: 10.1038/s41598-022-13569-4 (PMC9209431; doi:10.1038/s41598-022-13569-4)
Supplement: Supplementary file 2 — Supplementary Figures. [file 41598_2022_13569_MOESM2_ESM.pdf]

Figure S1

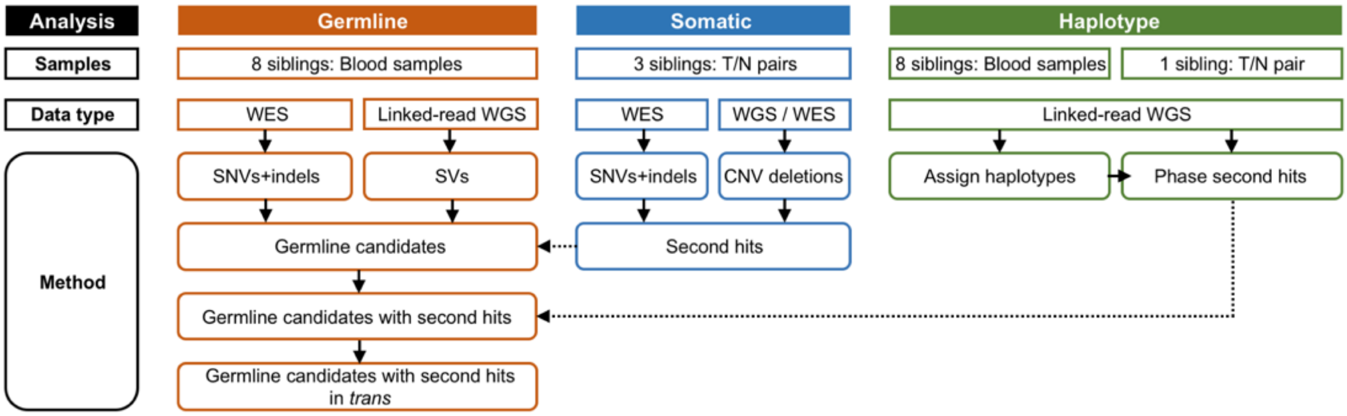

Figure S1. Overview of sequencing analysis methodology used to identify candidate germline variant in a family with CCA. Germline and somatic samples were sequenced with multiple sequencing technologies to detect variants. The resulting variants were integrated and filtered to identify a candidate causal mutation.

Figure S2

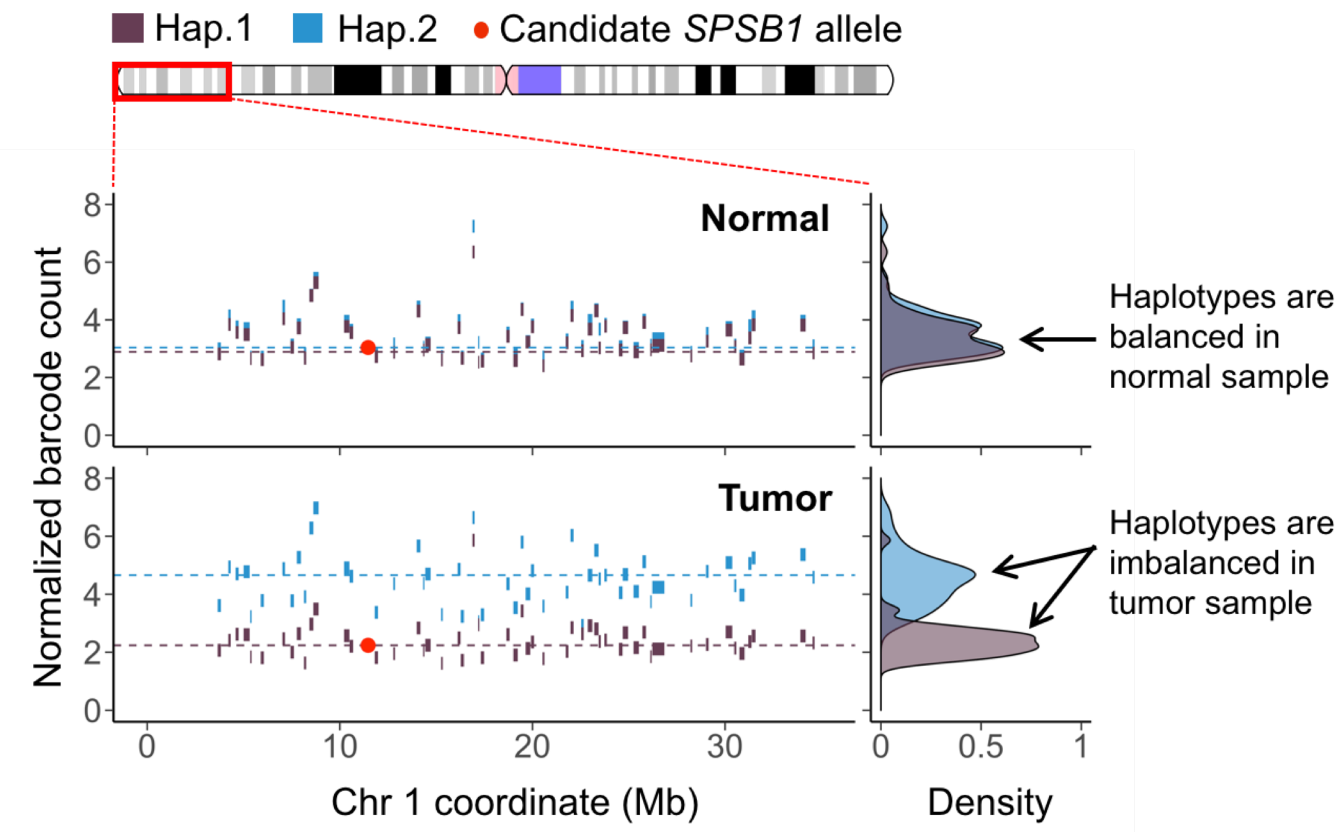

Figure S2. Extended haplotype of the 40 Mb deleted region of chromosome 1p in individual III:8. The blocks indicate the original fragmented haplotypes, and their color denotes their subsequent assignment to haplotypes covering many Mb. The candidate *SPSB1* allele exists in haplotype 1 (purple), which was the deleted haplotype in the tumor of this individual.

Figure S3

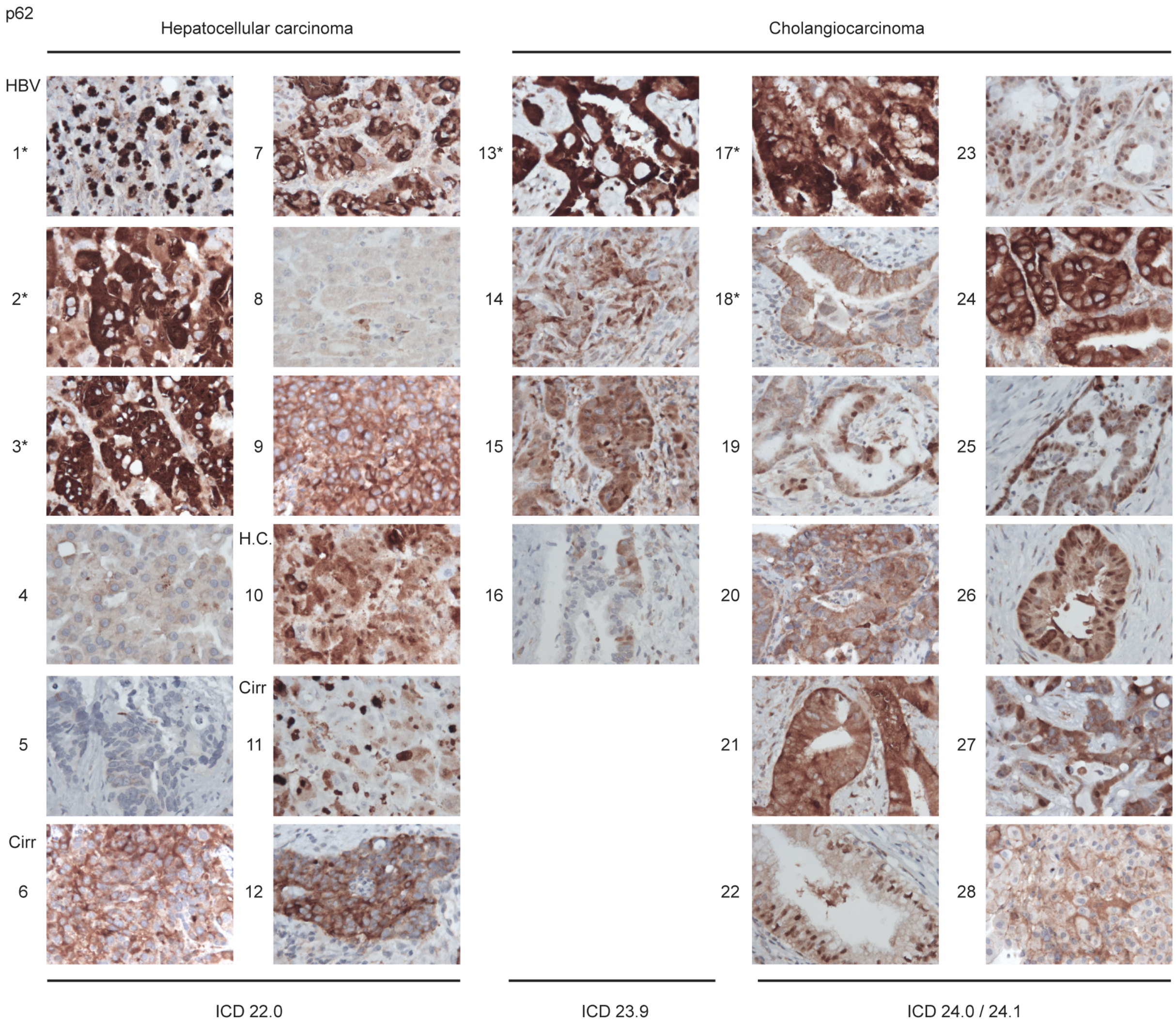

Figure S3. Increased p62 expression of HCC and CCA patients carrying the rs146589465-G variant. Sections from 28 paraffin-embedded HCC or CCA patient samples were stained with a p62 antibody. Numbers correlate with patient numbering in Table S20 and an asterisk indicates an rs146589465-G carrier. Information on liver disease is indicated; Cirr, cirrhosis; H.C., hemochromatosis; HBV, Hepatitis B virus. Samples are grouped by International Classification of Diseases (ICD) diagnoses information from the Icelandic Cancer Registry.

Figure S4

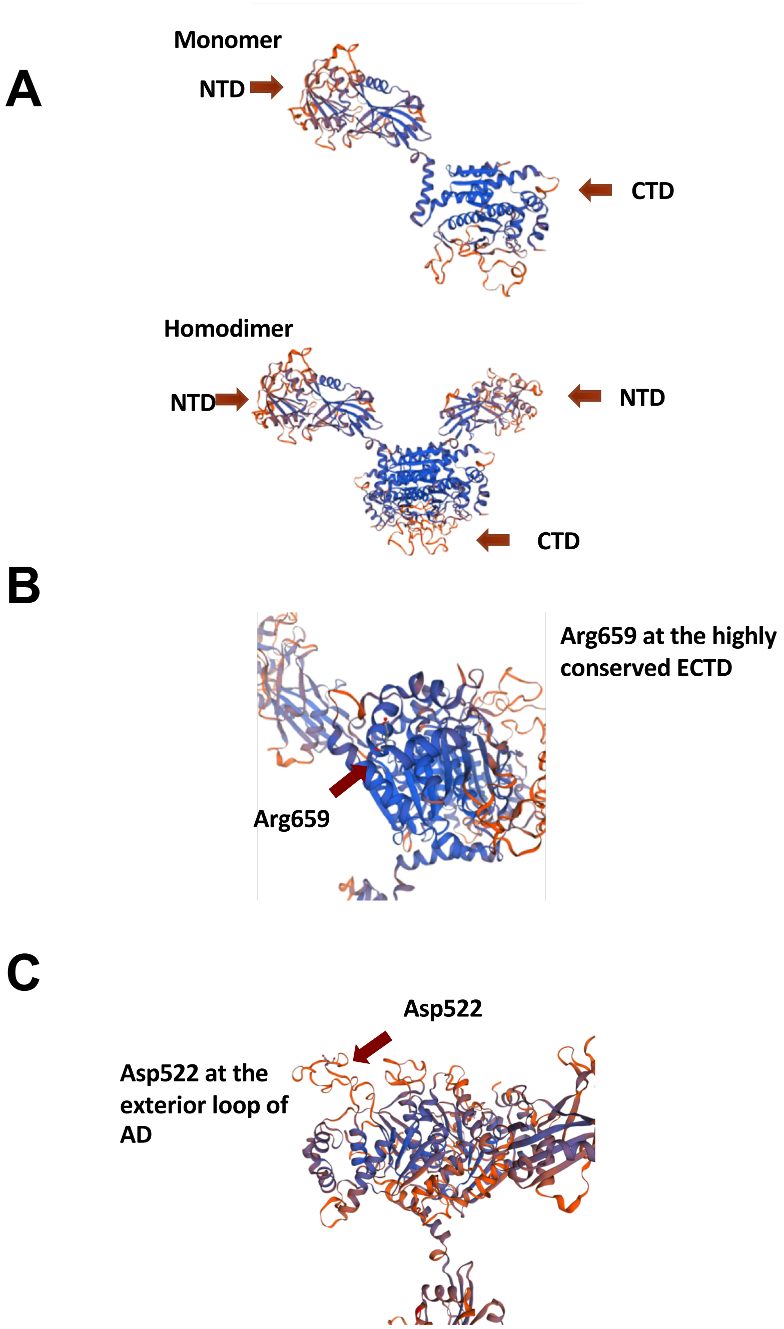

Figure S4. ATG7 protein structure and location of variants in the protein. (A) ATG7 functions as a homodimer. The locations of the CTD and NTD are indicated on the monomer and homodimer. **(B)** The Arg659 residue is located in the ECTD of ATG7. **(C)** The Asp522 residue is located in an exterior loop of the AD of ATG7.

Figure S5

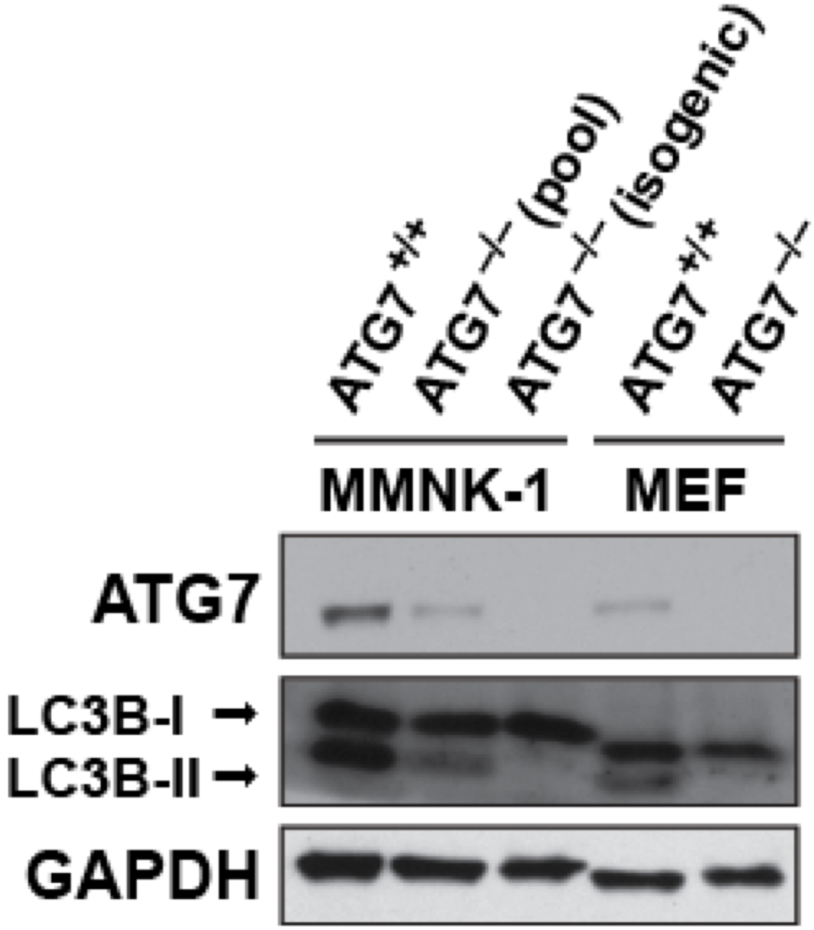

Figure S5. ATG7 p.R659* is a loss-of-function mutation. MMNK-1 ATG7$^{-/-}$ cells did not convert LC3B-I to the lipidated form LC3B-II. Mouse MEF Atg7$^{+/+}$ and MEF Atg7$^{-/-}$ were used as controls.

Figure S6

A

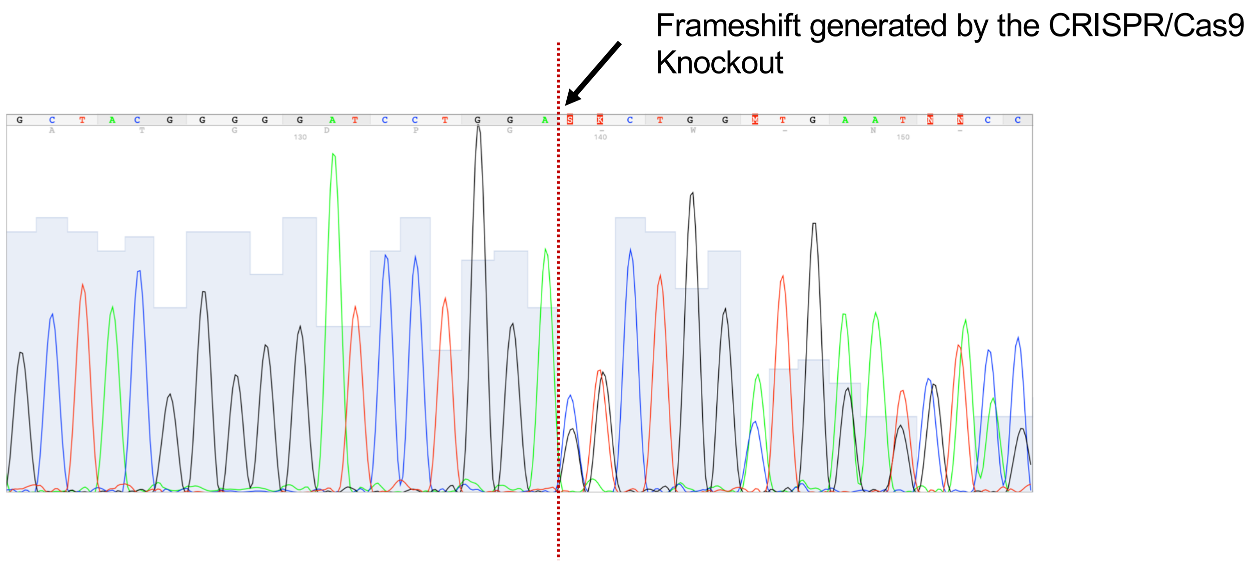

B

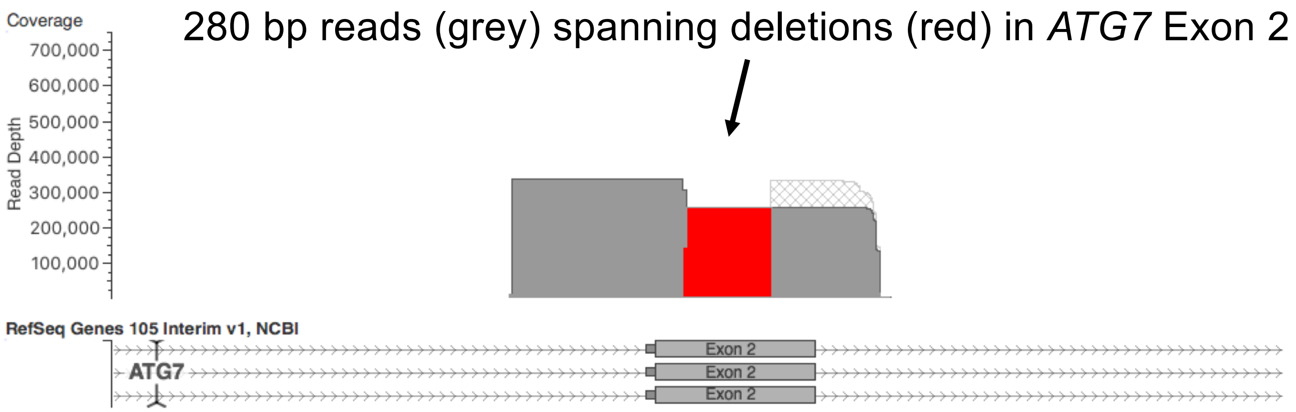

Figure S6. Confirmation of CRISPR deletion mutations in *ATG7* in the MMNK-1 cell line. (A) Sanger sequencing confirmed the presence of a deletion in an isogenic cell line. **(B)** Targeted amplicon sequencing with Illumina confirmed the presence of an 84-bp and 88-bp deletion in an isogenic MMNK-1 cell line (ATG7$^{-/-}$).

Figure S7

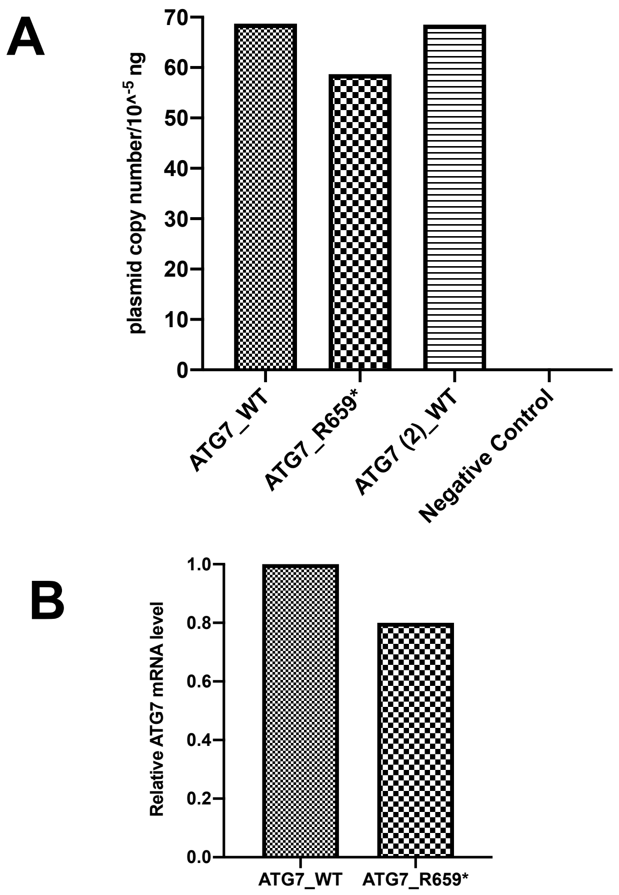

Figure S7. Quantification of plasmid input and mRNA expression level. (A) The copy number of each plasmid was quantified by ddPCR. The input amount of each plasmid was 10^{-5} ng/reaction. **(B)** Relative expression level of ATG7 mRNAs between WT and R659* expression in MMNK-1 ATG7$^{-/-}$. The housekeeping gene *RPP30* was used for normalization.

Figure S8

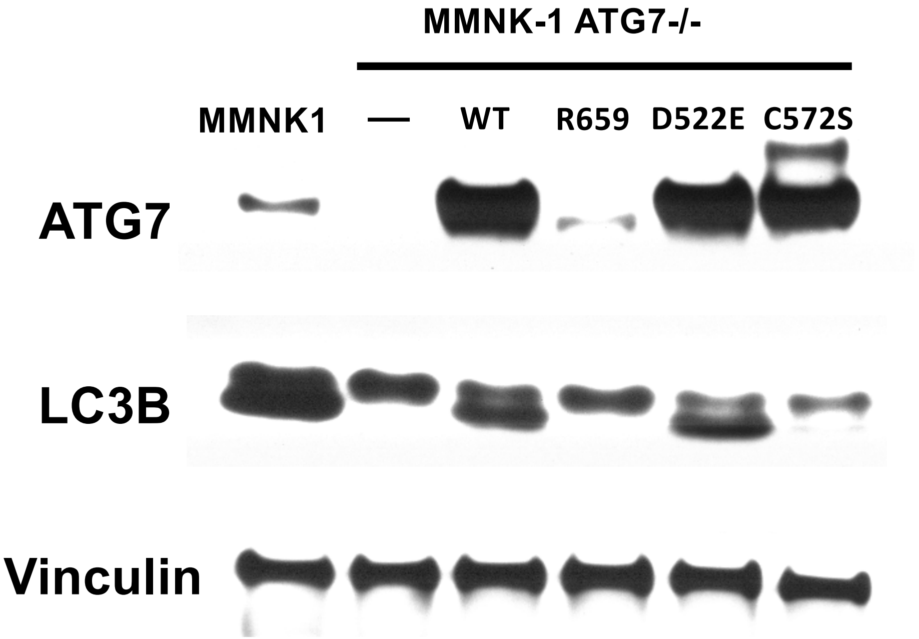

Figure S8. Expression of ATG7 in stably transduced MMNK-1 *ATG7$^{-/-}$* cells. Protein lysates are harvested from cells that were transduced and selected from lentivirus expressing ATG7 WT and mutants. As expected, R659* and C572S failed to lipidate LC3B.

Figure S9

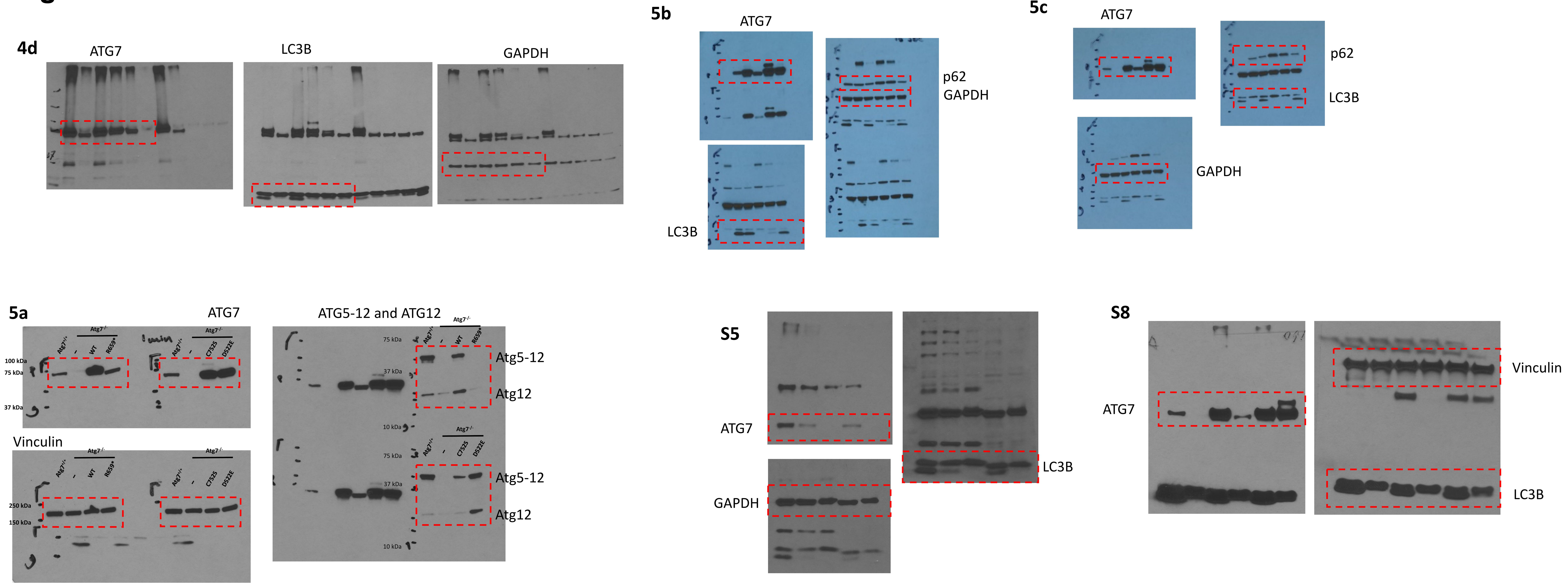

Figure S9. Full versions of western blots. Red dashed boxes indicate the blot sections cropped for each figure indicated.
